# Ostreid herpesvirus 1 latent infection and reactivation in adult Pacific oysters, *Crassostrea gigas*

**DOI:** 10.1016/j.virusres.2023.199245

**Published:** 2023-10-25

**Authors:** Konstantin Divilov, Xisheng Wang, Alexandra E. Swisher, Peyton C Yeoman, Maxwell Rintoul, Gary B. Fleener, Blaine Schoolfield, Chris Langdon, Ling Jin

**Affiliations:** a Department of Fisheries, Wildlife, and Conservation Sciences, Coastal Oregon Marine Experiment Station, Oregon State University, Hatfield Marine Science Center, Newport, OR 97365, USA; b Department of Biomedical Sciences, Carlson College of Veterinary Medicine, Oregon State University, Corvallis, OR 97331, USA; c Hog Island Oyster Co., Marshall, CA 94940, USA

**Keywords:** Pacific oysters, OsHV-1, Latent, Reactivation, qPCR, Nested PCR, Hemocytes

## Abstract

Ostreid herpesvirus 1 (OsHV-1) is one of the most economically important pathogens of Pacific oysters. Understanding the pathogenesis of this virus is critical to developing tools to control outbreaks on shellfish farms. OsHV-1 is genetically related to vertebrate herpesviruses, which have a lytic and a latent stage, with the latent stage capable of being reactivated to the lytic stage. Here, OsHV-1 latency in Pacific oysters was investigated in experimentally and naturally infected oysters. Lytic infection in one-year-old oysters injected with the Tomales Bay strain of OsHV-1 was detectable between 1 and 4 days post-injection (dpi) but was not detectable after 5 dpi. The injected oysters shed 1 × 10^2^ to 1 × 10^4^ DNA copies/ml into the water during the 4-day acute phase. Lytic shedding was not detectable in two-year-old oysters injected similarly with the same strain of OsHV-1; however, the OsHV-1 genome was detectable by qPCR in the adductor muscle, gill, mantle, and hemocytes within the first 3 dpi, after which it became undetectable. No OsHV-1 was detectable in the adductor muscle, gill, or mantle from experimentally infected oysters on days 15 and 21 post-injection or from oysters sampled 9 months after surviving an OsHV-1 mortality event; however, OsHV-1 DNA could be detected in hemocytes of both experimentally infected oysters at 21 dpi and naturally infected oysters using nested PCR. In addition, lytic viral gene transcription was detectable in hemocytes of experimentally infected oysters between 1 and 21 dpi and in hemocytes of naturally infected oysters. Furthermore, OsHV-1 reactivation from latency was induced in experimentally infected oysters at 21 dpi and in naturally infected oysters 12 months after an OsHV-1 outbreak.

## Highlights

• This is the first demonstration that a member of the *Malacoherpesviridae*, ostreid herpesvirus 1 (OsHV-1), becomes latent in the Pacific oyster (*Crassostrea gigas*) after primary infection.
• OsHV-1 latency can be detected in the hemocytes.
• OsHV-1 reactivation from latency can be induced by chemical and temperature stress.
• Sodium butyrate and TPCA-1 can induce OsHV-1 reactivation from latency alone or when combined together.
• OsHV-1 lytic gene transcription can be detected in some latently infected oysters.

## 1 Introduction

The Pacific oyster (*Crassostrea gigas*) is one of the world's most commonly cultivated shellfish (Botta, 2020). Ostreid herpesvirus 1 (OsHV-1) is a viral pathogen of Pacific oysters that has been implicated in numerous mortality outbreaks in this species (Fuhrmann et al., 2022). OsHV-1 is a member of the *Herpesvirales* order, which contains herpesviruses that infect and cause disease in a wide range of vertebrate and invertebrate animal hosts (Davison et al., 2005). The *Herpesvirales* order contains the *Alloherpesviridae, Orthoherpesviridae*, and *Malacoherpesviridae* families. Fish and amphibian herpesviruses belong to the *Alloherpesviridae* family; avian, mammalian, and reptilian herpesviruses belong to the *Orthoherpesviridae* family; and molluscan herpesviruses, including OsHV-1, belong to the *Malacoherpesviridae* family (Davison et al., 2009). The Malacoherpesviridae family comprises two genera with one species each (Lefkowitz et al., 2018), OsHV-1 and haliotid herpesvirus 1 (HaHV-1). Multiple variants of OsHV-1 exist with different levels of virulence in Pacific oysters (Agnew et al., 2020; Segarra et al., 2010; Trancart et al., 2022); however, the genetic basis of differences in virulence has yet to be determined.

One of the unique features of herpesviruses is a latent infection following an initial lytic infection (De Leo et al., 2020; Eide et al., 2011; Field et al., 2006; Jones, 2003; Knipe and Howley, 2013; Morgan et al., 2001; Stern et al., 2019). Upon initial infection of a susceptible host, the virus enters a lytic state where it replicates exponentially at the entry site and in peripheral tissues. Following the initial infection, the virus can enter a latent state in the surviving hosts in either peripheral nerve ganglion neurons or lymphatic cells (De Leo et al., 2020; Eide et al., 2011; Field et al., 2006; Jones, 2003; Knipe and Howley, 2013; Morgan et al., 2001; Stern et al., 2019). During latent infections, the viral genome usually is “hiding” in a few cells and remains dormant and therefore causes no harm to the infected host. However, the latent viral genome can be reactivated to produce infectious viral progeny under stress conditions, such as elevated temperature, injury, or immune suppression. Latency and reactivation have been well-studied in herpesviruses in the *Alloherpesviridae* and *Orthoherpesviridae* (Cohen, 2020; Hanson et al., 2011; Reed et al., 2014), but little is known about these processes in the *Malacoherpesviridae* despite their economic importance.

OsHV-1 latency has important implications for Pacific oyster aquaculture. Oysters are often moved around for different purposes, e.g., adults from the field are often moved into hatcheries as broodstock, and these transfers can potentially spread OsHV-1 unintentionally. Detecting latent infections can be difficult due to the small number of cells that are infected (Cohen, 2020; Reed et al., 2014). This can be a problem because a false negative can unintentionally introduce OsHV-1 into a hatchery or a bay. Currently, OIE-recommended methods for detecting OsHV-1, specifically qPCR of DNA extracted from oyster tissues (OIE, 2019), cannot detect latent infections. For example, Pernet and Petton (2015) found OsHV-1 lytic infection occurred in oysters when seawater temperature was suddenly raised to 21 °C (Pernet, 2015), even though the oysters had previously tested negative.

One way of increasing the likelihood of detecting latent infections is to test the tissue where OsHV-1 becomes latent and use more sensitive methods to detect low copy numbers of viral DNA. The predominate sites of latency of the *Alloherpesviridae* and *Orthoherpesviridae* are either neurons or immune cells (Cohen, 2020; Osterrieder et al., 2006; Reed et al., 2014); however, the location of latency for OsHV-1 has yet to be determined. In this study, OsHV-1 latency was investigated in both experimentally infected oysters and survivors of an OsHV-1 mortality event in the field using a more sensitive nested PCR, compared with the OIE qPCR assay, and a viral DNA isolation method was used to avoid contamination of viral DNA with host chromosomal DNA.

## 2 Materials and methods

### 2.1 Virus stock

OsHV-1 stocks were prepared using moribund oysters from a mortality event in Tomales Bay, California, in 2019, as previously described by Divilov et al. (2021). Whole oyster tissues were ground with a mortar and pestle in phosphate-buffered saline (PBS) at a 1:5 ratio (w/v). The homogenized tissues were first centrifuged at 4000 × g for 15 min at 4 °C, and then the supernatant was centrifuged at 10,000 × g at 4 °C for 30 min to remove extracellular organelles. The supernatant was then filtered through a 0.45 μm filter and saved as viral stocks at −80 °C. The absence of bacteria in the supernatant was confirmed by culturing the supernatant on a LB agar plate at 25 °C for 24 h. The viral concentrations of the viral stocks were estimated by the OIE OsHV-1 qPCR assay described below.

### 2.2 OIE OsHV-1 qPCR assay

Currently, standard OsHV-1 testing uses the quantitative PCR assay adopted by the World Organization for Animal Health (OIE), the intergovernmental organization responsible for improving animal health worldwide. The OIE OsHV-1 quantitative PCR (qPCR) with forward OsHV1BF, reverse B4 primers and a TaqMan probe (Table 1) developed by Martenot et al. (2010) was performed according to the manufacturer's instructions for the TaqMan Fast Advanced Master Mix (Thermo Fisher Scientific). Each reaction contained 5 µl of total DNA and was run for 40 cycles of 95 °C for 15 s, 48 °C for 30 s, and 68 °C for 30 s on a StepOne Real-Time PCR System (Thermo Fisher Scientific). To allow for the absolute quantification of OsHV-1 copies in samples, the PCR product was cloned into the pCR2.1-TOPO vector (Thermo Fisher Scientific) and confirmed via Sanger sequencing at Oregon State University's Center for Quantitative Life Sciences. Plasmids with the PCR product insert were then used to establish a standard curve using 10-fold serial dilutions from 10^8^ to 10 copies of the plasmid.

**Table 1:** Primer pairs used to detect DNA of OsHV-1 genes.

Name	Gene	Primer sequences (5′−3′)
	Transmembrane protein	
ORF88-F458		TAGTCGTGAAACCCACCACA
ORF88-R458		TCACATCACTTGGTGGCAAT
ORF88-F227		TGAGCGGTATTCCAACAACA
ORF88-R227		TAGGTGGAGGAGGAGCTTGT
	DNA polymerase	
ORF100-F368		TGTTTATGGCCGATTTGACA
ORF100-R368		TTGCGACAAGGAAGTCTGTG
ORF100-F170		AAAGACCGGTGCAAGAACTG
ORF100-R170		GATATGCACTGGCCGTCTTT
	Housekeeping	
EF1a_F:		GAGCGTGAACGTGGTATCAC
EF1a_R:		ACAGCACAGTCAGCCTGTGA
	OIE OsHV-1 qPCR	
Forward OsHV1BF		GTCGCATCTTTGGATTTAACAA
Reverse B4		ACTGGGATCCGACTGACAAC
Taqman probe		TGCCCCTGTCATCTTGAGGTATAGACAATC

### 2.3 Nested PCR developed for this study

The selection of primers for OsHV-1 sequence amplification was based on OsHV-1 genome sequence data available through Genbank (NC_005881). DNA polymerase and structural genes, such as membrane protein genes, are relatively conserved for a specific group of herpesviruses; therefore, nested primers specific for ORF100 and ORF88 (Table 1), which code for DNA polymerase and a transmembrane protein, respectively, were used to detect the OsHV-1 genome. PCR amplification was performed using a 25 µl solution consisting of 2.5 µl amplification buffer (10X PCR buffer with 15 mM MgCl2), 0.4 mM dNTP, 0.4 µM each primer, 1 U Taq (Thermo Fisher Scientific), and 20 ng–500 ng DNA sample. The mixture was subjected to 94 °C for 2 min, 35 cycles of 94 °C for 30 s, 55 °C for 30 s, and 72 °C for 30 s, followed by a 5 min elongation reaction at 72 °C after the final cycle. The nested PCR was performed using nested set primers (Table 1). A 2.5 µl aliquot of the first-run PCR product was included as a template in the second (nested) amplification under the same PCR running conditions. A 1 Kb Plus DNA Ladder (Thermo Fisher Scientific) served as size markers in gel electrophoresis.

### 2.4 Oyster infection and viral reactivation from latency

Adult Pacific oysters, one or two years old (∼7 cm or 14 cm in shell length, respectively), were obtained from Yaquina Bay, Oregon, and brought to a BSL-2 laboratory in the Carlson College of Veterinary Medicine at Oregon State University Corvallis, Oregon. Larvae, spat, and young adult oysters from this bay have been tested annually for the presence of OsHV-1 via qPCR testing conducted by a USDA-APHIS accredited diagnostic laboratory (AquaTechnics Inc.). No positive tests or outbreaks had occurred in this bay; therefore, oysters from this bay were considered to be free of OsHV-1.

**Fig. 1:**
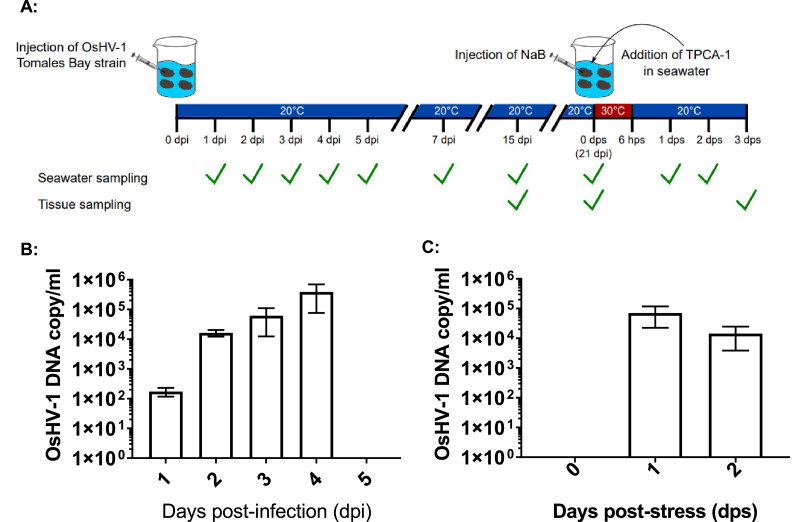
Schematic of OsHV-1 infection, reactivation, and detection of OsHV-1 by quantitative PCR (qPCR). **A**: Schematic of OsHV-1 infection in 1-year-old oysters obtained from Newport, Oregon. The oysters were injected with 100 µl of 1 × 10^7^ DNA copy/ml via adductor muscle injection and maintained at room temperature (20–22 °C). Oysters that survived the infection were maintained at room temperature and stressed at 21 days post-injection (dpi) as described in the Materials and methods. The check marks represent the seawater and tissue sampling dates.  **B**: qPCR of OsHV-1 DNA detected in the seawater samples (*n* = 4) from the container kept all the injected oysters collected at 1, 2, 3, 4, 5, 7, 15, and 21 dpi (Fig. 1A). **C**. qPCR of OsHV-1 DNA in the seawater samples (*n* = 3) from 6 injected oysters kept together in 1 L seawater collected before (0) and after stress at 1 and 2 days post-stress (dps).

Oysters were acclimatized in seawater for 5–7 days at 14–16 °C after arrival. Before infection, oysters were anesthetized overnight at room temperature (20–22 °C) in an aquarium filled with Coral Pro Salt (Red Sea) seawater containing 50 g/L MgCl_2_. The next day, 15–20 oysters were injected with 100 μl of an OsHV-1 (Tomales Bay strain) stock at 1 × 10^7^ OsHV-1 DNA copies/ml into the adductor muscle of each oyster with a 1 ml syringe using a 27-gauge needle and returned to the aquarium 30 min after injection. All injected oysters were kept together in about 3.5 L of seawater in a 9 L container. Another ten aged-matched oysters without OsHV-1 injection were kept together in a separate container in a similar amount of seawater as a control. For the next 21 days, oysters were kept in seawater at room temperature (20–22 °C) and fed *ad libitum* with LPB Frozen Shellfish Diet (Reed Mariculture). The aquarium water was changed every 7 days. Seawater samples were collected post-injection on days 1–5, 7, 15, and 21 to monitor virus shedding, as indicated in Fig. 1A.

To induce OsHV-1 reactivation from latency, 6 oysters at 21 days post-infection (dpi) were relaxed in 50 g/L MgCl_2_ overnight and injected with 100 µl of sodium butyrate (125 mg/ml) (Cat No. B5887, Sigma-Aldrich) per oyster. The injected oysters were kept together in 1 L of seawater containing 1 µM TPCA-1 (Cat No. T1452, Sigma-Aldrich) at 30 °C for 6 h before returning the stressed oysters to seawater at room temperature (Fig. 1A). Three infected oysters that were not stressed at 21 dpi were kept together in a separate container as a control.

### 2.5 OsHV-1 shedding into seawater

Seawater samples were collected with three or four replicates per time point (Fig. 1). According to the manufacturer's instructions, viral DNA was isolated from 200 µl of each seawater sample using the QIAamp DNA Blood Mini Kit (QIAGEN). The OIE OsHV-1 qPCR assay was then used to quantify viral load in the seawater samples. Viral DNA detected in 200 µl of seawater sample was converted to viral genome copy number per ml.

### 2.6 OsHV-1 detection and quantification in tissues

Total DNA of tissues was isolated by first homogenizing 0.1–0.2 g tissue in 400 µl of tissue lysis buffer from the EZNA Tissue DNA Kit (Omega Bio-tek), then extracted according to the manufacturer's instructions for the EZNA Tissue DNA Kit. Total DNA was eluted in 100 µl of TE buffer (10 mM Tris-HCL [pH 8.0]−1 mM EDTA) and then quantified using a NanoDrop spectrophotometer (Thermo Fisher Scientific). Approximately 500 ng of DNA per tissue was used to perform the OIE OsHV-1 qPCR assay. Viral DNA detected in tissue was reported as viral genome copy number per µg of total tissue DNA.

### 2.7 DNA sequencing of PCR product

PCR or reverse-transcription-polymerase chain reaction (RT-PCR) products were purified using a ChargeSwitch PCR Clean-Up kit (Invitrogen), and the cleaned PCR product was directly sequenced by the Center for Quantitative Life Sciences at Oregon State University. The nucleotide sequences were analyzed with the Geneious Prime 2023 software.

### 2.8 OsHV-1 detection and quantification in hemocytes

One to two ml of hemolymph was drawn from each oyster's adductor muscle with a 1 ml syringe fitted with a 27 gauge needle. The hemolymph was diluted in 3 vol of 1X PBS and centrifuged at 4000 × g for 10 min to pellet the hemocytes. The cell pellet was washed once in 1X PBS and then resuspended in 400 µl of hypotonic buffer (50 mM Tris–HCl pH 8.0, 5 mM EDTA, 50 g/ml RNAse, 2 % Triton X-100) at 4 °C for 24 h (19). Cell nuclei from the post-hypotonic treatment were pelleted by centrifugation at 10,000 × g for 10 min at 4 °C. The supernatant was loaded directly to the HiBind DNA Mini Column (Omega Bio-tek). The DNA binding column was washed according to the manufacturer's instructions for the EZNA Tissue DNA Kit, and DNA was eluted in 50 µl of TE buffer. Around 10–50 ng of DNA isolated from the supernatant was used in the PCR for detection of OsHV-1. Total DNA from the pellet of the hypotonically-treated cell nuclei was isolated according to the manufacturer's instructions for the EZNA Tissue DNA Kit, and the DNA was also eluted in 50 µl of TE buffer. Approximately 100–500 ng of DNA isolated from the cell pellet was used in the PCR.

### 2.9 OsHV-1 gene expression

Total RNA was extracted from ∼1 × 10^7^ oyster hemocytes using the High Pure RNA Isolation Kit (Roche). cDNA was synthesized with the qScript cDNA Synthesis Kit (Quantabio) and used as a template for the detection of mRNA of ORF100 and ORF88 (Table 1). Three μl of cDNA were used as a template in the first run of the nested PCR assay described above. A 2.5 µl aliquot of the original PCR product was included as a template in the second (nested) amplification. RT-PCR with and without reverse transcription was also performed with primers EF1a_F and EF1a_R specific to the *C. gigas* housekeeping gene EF1a (elongation factor 1-alpha) (LOC105338957) to ensure that comparable levels of input RNA were used in RT-PCR and to ensure that amplification did not originate from residual DNA. The RT-PCR product of EF1a is expected to be at 143 bp from processed mRNA and 228 bp from genomic DNA.

### 2.10 OsHV-1 persistence and reactivation in Pacific oysters surviving a mortality event in the field

Pacific oyster spat were planted in Tomales Bay, California, in April 2022 for the purpose of commercial production. Those growing oysters experienced an OsHV-1 mortality event in July 2022. Survivors of this mortality event were kept in the bay until they were harvested in April 2023 and shipped overnight on ice to a BSL-2 laboratory in the Carlson College of Veterinary Medicine at Oregon State University in Corvallis, Oregon. To investigate OsHV-1 persistency, adductor muscle, gill, mantle tissue, and hemocytes were collected from these oysters upon arrival. DNA and RNA were then extracted from these tissues and hemocytes to conduct the qPCR and nested PCR assays described above. An additional set of oysters from the same planting were harvested in July 2023 and shipped overnight on ice. Oysters were acclimatized in seawater for five days at 14–16 °C after arrival, after which induction of OsHV-1 reactivation was carried out using the following two methods. In the first method, ten oysters were relaxed in 50 g/L MgCl2 overnight, injected with 100 µl of sodium butyrate (125 mg/ml) (Cat No. B5887, Sigma-Aldrich), and then kept together in a 3.5 L aquarium containing seawater at room temperature., In the second method, ten oysters were kept in a 3.5 L aquarium containing seawater, with 1 µM TPCA-1 added, at room temperature. Another ten oysters were not stressed and kept together in a 3.5 L aquarium containing seawater at 14–16 °C as a control. Gill tissue from three oysters was examined for the presence of OsHV-1 upon arrival, before stress, and 24 h after stress.

## 3 Results

### 3.1 OsHV-1 reactivation in experimentally infected one-year-old adult oysters at 21 days post-infection

OsHV-1 lytic shedding in seawater immediately after injection was examined from 20 injected oysters kept together in 3.5 L of seawater at 20 °C (Fig. 1A). Lytic virus shedding into the seawater was monitored daily within the first 5 days post-injection, then at 7, 15 and 21 day post-injection. OsHV-1 DNA copy number was estimated by the OIE-approved TaqMan qPCR assay in three aliquots of 200 µl of seawater from the tanks. As shown in Fig. 1B, OsHV-1 lytic shedding was detected at 1, 2, 3, and 4 dpi with mean OsHV-1 copies of 1.9 × 10^2^, 1.1 × 10^4^, 6.1 × 10^4^, and 2.2 × 10^5^ OsHV-1 DNA copies/ml, respectively. The total amount of virus shed in the tank ranged between 7 × 10^5^ to 8 × 10^8^ OsHV-1 DNA copies within the first 4 dpi. No OsHV-1 shedding was detected on 5, 7, 15 and 21 dpi. No OsHV-1 was detected in DNA samples extracted from adductor muscle, gill, intestine, labial palp, or mantle tissues collected from three oysters at 15 and 21 dpi. No mortality was observed in the group of oysters injected with OsHV-1. Similarly, no mortality was observed in uninfected oysters. No OsHV-1 shedding was detected in the seawater of control oysters.

If OsHV-1 becomes latent in these surviving oysters, OsHV-1 shedding in seawater after stress will suggest OsHV-1 reactivation from latency. Herpesvirus reactivation from latency can be induced by immune suppression (Alfaro et al., 2023; Kampouri et al., 2023) and histone chromatin modification (Danaher et al., 2005; Hopcraft et al., 2018). Sodium butyrate (NaB) is a histone deacetylase inhibitor and can induce HSV-1 reactivation from latency in mouse models (Neumann et al., 2007). TPCA-1 is an NF-kB innate immune pathway inhibitor and can block inflammatory responses (Larner-Svensson et al., 2010). OsHV-1 shedding in seawater was again examined in six oysters treated with NaB and TPCA-1 and kept together in 1 L seawater at 21 dpi. As shown in Fig. 1C, OsHV-1 lytic shedding at 7.0 × 10^4^ and 1.7 × 10^4^ OsHV-1 DNA copies/ml were again detected at 1 and 2 days post-stress (dps), respectively. To further confirm OsHV-1 reactivation from latency, all oysters were harvested on 3 dps. OsHV-1 was detected in the adductor muscle, gill, and mantle from two of the six oysters, with OsHV-1 copies/µg ranging from 4903 to 9931 (Table 2). No OsHV-1 was detected in all three infected oysters that were not stressed. These results suggest OsHV-1 can be reactivated from latency under stress conditions.

**Table 2:** OsHV-1 detection and quantification tissues at 3 days post-stress from six oysters.

Oyster	Adductor muscle	Gill	Mantle
1	−	−	−
2	−	−	−
3	−	+ (7669)	+ (6400)
4	+ (9931)	+ (4903)	−
5	−	−	−
6	−	−	−

*Note:* Viral DNA (OsHV-1 copies/μg total DNA) are given in parentheses for positive samples.

### 3.2 OsHV-1 latency in experimentally infected two-year-old adult Pacific oysters

**Fig. 2:**
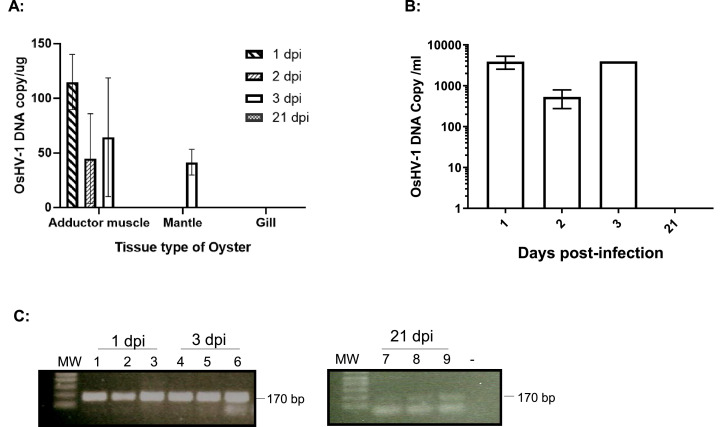
Detection of OsHV-1 in total DNA of oyster tissues and hemocytes in injected two-year-old oysters. **A**: OsHV-1 DNA detected by qPCR in tissues of oysters at 1, 2, and 3 dpi. **B**: qPCR of OsHV-1 DNA in hemocytes at 1, 2, 3, and 21 dpi. **C**: Detection of OsHV-1 DNA by nested PCR in hemocytes collected at 1, 3, and 21 dpi. Hemocytes were collected from three injected oysters at 1, 3, and 21 dpi. Viral DNA was extracted from the supernatant of hemocytes after a hypotonic buffer treatment. The nested PCR was performed with PCR primers OsHV-1–368F and OsHV-1–368R in the first run and PCR primers OsHV-1–170F and OsHV-1–170R in the second run. PCR products amplified from the second run were expected to be 170 bp. Lanes 1–3: DNA extract of hemocytes from oysters 1–3 collected at 1 dpi; Lanes 4–6: DNA extract of hemocytes from oysters 4–6 collected at 3 dpi; Lanes 7–9: DNA extract of hemocytes from oysters 7–9 collected at 21 dpi. N: negative control. MW: 1 Kb Plus DNA Ladder (Thermo Fisher Scientific).

To investigate the latency sites of OsHV-1 infection, 15 two-year-old oysters, ∼14 cm in shell length, were infected similarly as above. OsHV-1 shedding in the seawater was monitored daily for 3 dpi, as described above. No OsHV-1 shedding was detectable by qPCR in the seawater at 1, 2, and 3 dpi. To prove acute infection occurred in the infected oysters, adductor muscle, gill, and mantle tissues were collected from three oysters at 1, 2 and 3 dpi. OsHV-1 at concentrations of 20 to 133 copies/μg were detected in total DNA samples of adductor muscle and mantle tissues, but no OsHV-1 was detected in the total DNA samples of the gill tissues (Fig. 2A). In addition, viral DNA at 280 to 5450 OsHV-1 copies/ml were detected in hemolymph samples within the first 3 dpi (Fig. 2B); however, no OsHV-1 DNA was detectable by qPCR in the total DNA of tissues or hemocytes tested on 21 dpi.

**Fig. 3:**
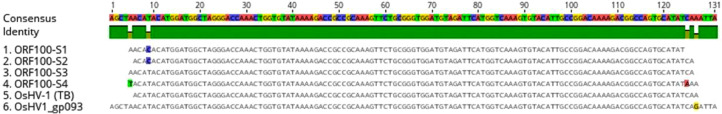
DNA sequence alignments of nested PCR and RT-PCR products amplified with primers specific ORF100. ORF100 DNA sequence alignment with the OsHV-1 reference genome's (AY509253) ORF100 gene (OsHV1_gp093). ORF100-S1 and ORF100-S2: Nested PCR product amplified from hemocyte DNA of oysters 1 and 4 at 1 and 3 dpi, respectively (Fig. 2). ORF100-S3 and ORF100-S4: Nested RT-PCR product amplified from total RNA of hemocytes isolated from oysters 2 and 4 (Fig. 7B, lanes 2 and 4) collected from mortality event survivors in Tomales Bay. OsHV-1 (TB): OsHV-1 (Tomales Bay strain).

To determine whether hemocytes might be the sites of OsHV-1 latency, DNA was isolated from hemocytes harvested at 21 dpi by treating them with a hypotonic buffer to reduce host nuclear DNA (Jin et al., 2004). The presence of OsHV-1 DNA in the supernatant of hemocytes after hypotonic buffer treatment was examined by OIE qPCR and nested PCR with primers specific to ORF100, which encodes the DNA polymerase (Table 1). The qPCR can detect OsHV-1 DNA within the first 3 days post-infection but failed to detect OsHV-1 in hemocytes tested on 21 dpi (Fig. 2B); however, as shown in Fig. 2C, a correct-sized fragment, 170 bp, was amplified by nested PCR from hemocytes of all three tested oysters on 1, 3 and 21 dpi, although the amplification was weak on 21 dpi (Fig. 2C). DNA sequences of the PCR product from oyster one at 1 dpi (Fig. 2C lane 1), labeled as ORF100-S1 in Fig. 3, and oyster four at 3 dpi (Fig. 2C, lane 4), labeled as ORF100-S2 in Fig. 3, are perfectly aligned with the PCR product of the Tomales Bay strain of OsHV-1 (TB) (Fig. 3). This result suggests that the OsHV-1 viral genome persists in hemocytes of oysters after acute infection.

### 3.3 OsHV-1 lytic gene expression persists in hemocytes of oysters

**Fig. 4:**
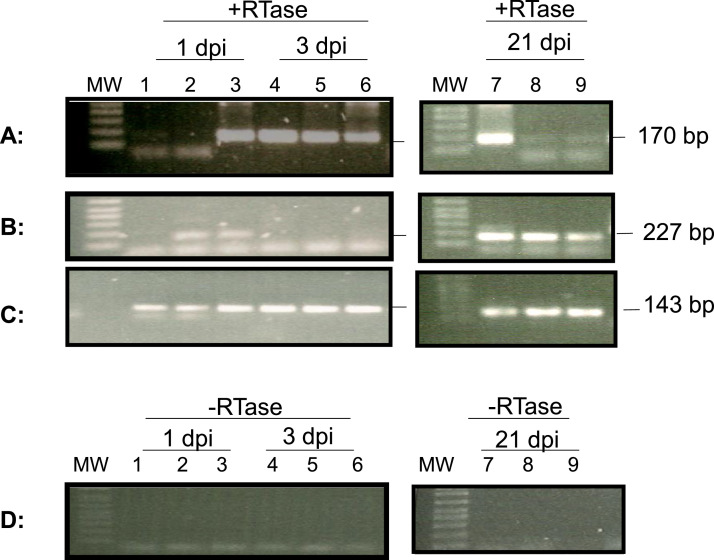
Detection of OsHV-1 lytic transcripts in hemocytes at 1, 3, and 21 dpi. Total RNA was isolated from three injected oysters collected on 1, 3, and 21 dpi. The cDNA synthesized by random primers from the total RNA in the presence of reverse transcriptase (+RTase) and absence of reverse transcriptase (-RTase) was amplified with primers specific to ORF100 (A and D) and ORF88 (B), and the internal control oyster gene, elongation factor 1-alpha (EF1a) (C).  Lanes 1–3: total RNA of hemocytes from oysters 1–3 on 1 dpi; Lanes 4–6: total RNA of hemocytes from oysters 4–6 on 3dpi; Lanes 7–9: total RNA of hemocytes from oysters 7–9 on 21dpi. MW: 1 Kb Plus DNA Ladder (Thermo Fisher Scientific). The ORF100, ORF88, and EF1a PCR products are expected to be 170 bp, 227 bp, and 143 bp, respectively.

To determine whether lytic viral gene transcription occurred in the hemocytes during the acute phase of infection, lytic gene transcription was examined in the total RNA isolated from 1 to 2 ml of hemocytes of three infected two-year-old oysters on 1 and 3 dpi. The cDNA synthesized from the total RNA of hemocytes was analyzed with primers specific for the DNA polymerase gene (ORF100) and a transmembrane gene (ORF88), respectively (Burioli et al., 2017; Davison et al., 2005). As shown in Fig. 4, ORF100 mRNA was detected by RT-PCR in hemocytes from one out of three oysters on 1 dpi and from all three oysters on 3 dpi. Similarly, mRNA of ORF88 was detected by RT-PCR in hemocytes from two out of three oysters at 1 dpi and from none of the three oysters at 3 dpi (Fig. 4). These results indicated lytic viral gene transcripts occur in the hemocytes during lytic infection phase.

To determine if lytic viral gene transcription was still present at 21 dpi in these infected two-year-old oysters, total RNA was also isolated similarly from hemocytes of three oysters at 21 dpi. As shown in Fig. 4A aand Fig. 4B, mRNA from both ORF100 and ORF88 were detectable in all three oysters sampled at 21 dpi. The amplification signal at 21 dpi was similar to that detected during the acute phase. The amplification of the housekeeping gene EF1a was performed as an internal control to ensure that comparable levels of input RNA were used in RT-PCR. EF1a amplification was similar from all three oyster hemocytes tested on 1, 3, and 21 dpi (Fig. 4C). No ORF100 amplification was detected by RT-PCR without reverse transcriptase in the reaction using total RNA extracted from hemocytes at 1, 3, and 21 dpi (Fig. 4D). No ORF88 and EF1a amplification was detected either by RT-PCR without reverse transcriptase in the reaction using total RNA extracted from hemocytes at 1, 3, and 21 dpi, respectively (Supplemental Fig. 1).

### 3.4 OsHV-1 persistence in hemocytes of Pacific oysters surviving a mortality event in the field

**Fig. 5:**
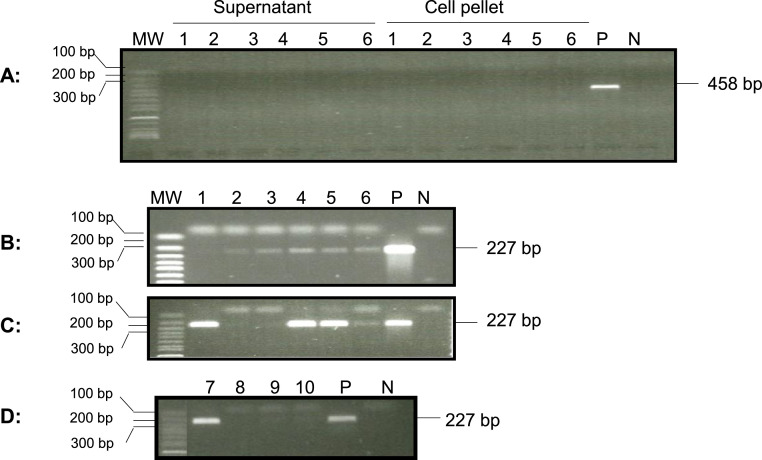
Detection of OsHV-1 in hemocytes of oysters that survived a mortality event in Tomales Bay, California. The nested PCR was performed with PCR primers ORF88-F458 and ORF88-R458, in the first run (**A**) and PCR primers ORF88-F227 and ORF88-R227, in the second run with DNA extracted from the supernatant (**B**) or cell pellet (**C**) of hemocytes after a hypotonic buffer treatment, and total DNA of hemocytes isolated by EZNA Tissue DNA Kit (**D**). Lanes 1 to 6: hemocyte DNA from oysters 1 to 6. Lanes 7–10: hemocytes DNA from oysters 7 to 10. N: negative control; P: OsHV-1 Tomales Bay strain DNA. MW: 1 Kb Plus DNA Ladder (Thermo Fisher Scientific). The ORF88 PCR products are expected to be 458 bp in the first run and 227 bp in the second run, respectively.

To determine whether OsHV-1 was still present in one-year-old oysters that survived a summer mortality event in Tomales Bay, adductor muscle, gill, and mantle tissues from four randomly selected oysters were screened for the OsHV-1 genome by OIE qPCR. No OsHV-1 DNA was detected by qPCR in the total DNA of tissues collected from those four oysters. To determine whether OsHV-1 was present in oyster hemocytes, the supernatant DNA from the post-hypotonic buffer treatment from another 6 oysters was examined by nested PCR with a primer specific to ORF88. A correct-sized fragment, 227 bp, was amplified by nested PCR from five out of six oyster hemocyte supernatants from the post-hypotonic buffer treatment (Fig. 5B, lanes 1 to 6). Two of the six hemocyte supernatants also tested positive for OsHV-1 by qPCR. In addition, OsHV-1 was also detectable in four out of six hemocyte DNA samples isolated from the cell pellets after post-hypotonic buffer treatment (Fig. 5C).

**Fig. 6:**
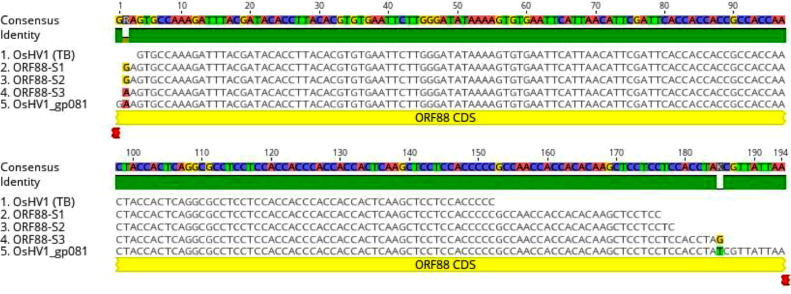
DNA sequence alignments of nested PCR and RT-PCR products amplified with primers specific to ORF88. ORF88 DNA sequence alignment with the OsHV-1 reference genome's (AY509253) ORF88 gene (OsHV1_gp081). ORF88-S1 and ORF88-S2: Nested PCR product amplified from DNA isolated from oyster 1 and 4 hemocytes (Fig. 5C, lanes 1 and 4), respectively, ORF88-S3: Nested RT-PCR product amplified from total RNA isolated from oyster two hemocytes (Fig. 7A, lane 2). OsHV-1 (TB): OsHV-1 Tomales Bay strain.

To determine if OsHV-1 can be detected by nested PCR from total DNA of hemocytes without hypotonic buffer treatment, total DNA of hemocytes were isolated by the EZNA Tissue DNA Kit, and tested by nested PCR with primers specific to ORF88. As shown in Fig. 5D, only one of the four samples tested positive. These results demonstrated that detection is more sensitive using DNA isolated from the supernatant of the post-hypotonic buffer treatment for OsHV-1 in hemocytes. To confirm that the amplified products were indeed OsHV-1 DNA, the nested-PCR products from oysters 1 and 4 (Fig. 5C, lanes 1 and 4), labeled as ORF88-S1 and ORF88-S2 in Fig. 6, respectively, were sequenced directly. As shown in Fig. 6, ORF88-S1, and ORF88-S2 have 100 % homology with the Tomales Bay strain of OsHV-1 (Fig. 6, 88-OsHV-1). The above result confirms that OsHV-1 persists in the hemocytes of naturally infected oysters.

**Fig. 7:**
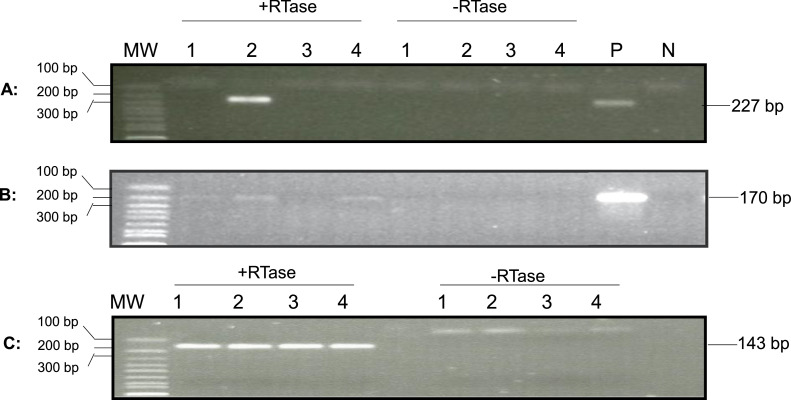
Detection of OsHV-1 lytic gene transcripts in hemocytes by nested PCR. Total RNA was isolated from four oysters from Tomales Bay, California, that survived a summer OsHV-1 mortality event. The cDNA synthesized by random primers from the total RNA in the presence of reverse transcriptase (+RTase) and absence of reverse transcriptase (-RTase) was amplified with primers specific to ORF88 (A), ORF100 (B), and EF1a (C). Lanes 1–4: total RNA of hemocytes from oysters 1–4. P: OsHV-1 Tomales Bay strain DNA. N: negative control. MW: 1 Kb Plus DNA Ladder (Thermo Fisher Scientific).

To determine whether lytic viral gene transcription was still present in the hemocytes of these naturally infected oysters, viral lytic gene transcription was examined in the total RNA isolated from 1 to 2 ml of hemocytes from another four oysters. The synthesized cDNA made from the total RNA of hemocytes was analyzed with primers specific for the DNA polymerase gene (ORF100) and transmembrane gene (ORF88) as described above. As shown in Fig. 7, ORF88 mRNA was detected by nested RT-PCR in hemocytes from one of the four oysters (Fig. 7A), and the mRNA of ORF100 was detected in three of the four tested oysters (Fig. 7B). All four samples had similar amplification with primers specific to a host gene, EF1a, used as an internal loading control (Fig. 7C). No product was amplified with cDNA from RT without reverse transcriptase (Fig. 7C, lanes 1–4 under -RTase). To confirm that the amplified products were indeed OsHV-1 DNA, sequencing was carried out from the nested RT-PCR products of ORF88 in Fig. 7A, lane 2 (+RTase) labeled as ORF88-S3 in Fig. 6, and ORF100 in Fig. 7B, lanes 1 and 2 (+RTase) labeled as ORF100-S3 and ORF100-S4 in Fig. 3, respectively. As shown in Figs. 3 and 6, the DNA sequence of the nested RT- PCR products of ORF88 and ORF100 are almost identical to the OsHV-1 reference gene sequences OsHV1_gp081 (ORF88) and OsHV1_gp093 (ORF100), respectively. These results indicate that lytic gene transcription exists in some latently infected oysters.

### 3.5 OsHV-1 reactivation from latency in Pacific oysters surviving a mortality event in the field

**Fig. 8:**
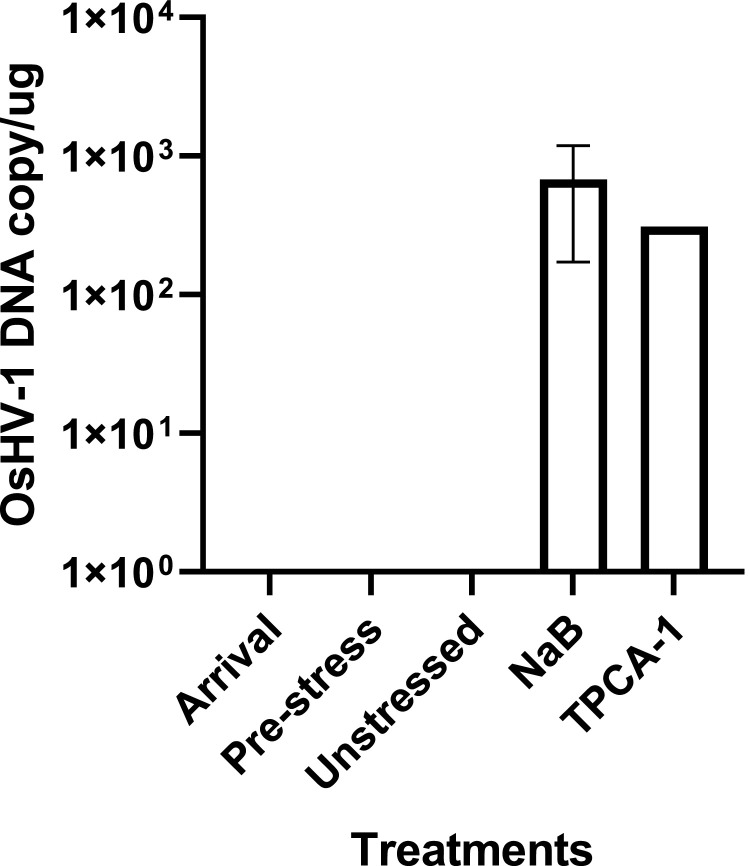
OsHV-1 qPCR using total DNA of gill tissues (*n* = 3 samples per treatment) collected from oysters upon arrival, before stress, after stress, and without stress. Arrival: gill DNA obtained from oysters sampled upon arrival from Tomales Bay, California. Pre-stress: gill DNA obtained from oysters before stress. Unstressed: gill DNA obtained from oysters kept at 14–16 ºC without any stress treatment. NaB: gill DNA obtained from oysters 24 h after being injected with sodium butyrate and kept at room temperature. TPCA-1: hemocyte DNA obtained from oysters 24 h after being kept in seawater containing 1 uM of TPCA-1 at room temperature.

To determine whether OsHV-1 reactivation from latency can be induced from naturally infected oysters that survived a mortality event in Tomales Bay, California, in July 2022, OsHV-1 reactivation from latency was examined in gill tissues from three randomly selected oysters collected in July 2023, and stressed with either NaB or TPCA-1 alone as described above. As shown in Fig. 8, around 1 × 10^2^ and 6 × 10^3^ OsHV-1 DNA copy/µg can be detected in gills at 1 dps from 2 out 3 oysters stressed with NaB and in hemocytes at 1 dps from 1 out 3 oysters stressed with TPCA-1, respectively. No OsHV-1 was detected in the gills of oysters upon arrival or prior to the application of stress, i.e., NaB or TPCA-1 (Fig. 8). Additionally, no OsHV-1 was detected in the gills of oysters that were not stressed (Fig. 8). This result demonstrates that oysters that survived the mortality event are latently infected with OsHV-1, and OsHV-1 can be reactivated by chemical stress or immune suppression.

## 4 Discussion

Shedding of OsHV-1 into the seawater is a sign of a lytic OsHV-1 infection in Pacific oysters. Upon injection of one-year-old Pacific oysters with OsHV-1, the presence of OsHV-1 viral genome in the seawater was observed for the first four days but was not observed between 5 and 21 dpi, which suggests a brief acute infection occurred in the injected oysters. No OsHV-1 DNA was detected in tissues of those injected oysters at 15 and 20 dpi, which means OsHV-1 lytic infection is cleared from the infected host. Although no OsHV-1 was detected in tissues, such as gills, mantle, and abductor muscle after the lytic infection, a low copy of OsHV-1 DNA was detectable in the hemocytes from both experimentally infected oysters at 21 dpi (Fig. 3). Similarly, OsHV-1 was also found to be present in hemocytes of oysters sampled 9 months after surviving an OsHV-1 mortality event in Tomales Bay (Fig. 5). These results suggest that OsHV-1 can become latent in the infected oysters. In agreement with our finding, the persistence of OsHV-1 after mortality events has been reported in other locations; for example, OsHV-1 was found in survivors from OsHV-1 mortality events in La Cruz lagoon, Mexico; Marennes-Oléron Bay, France; and Woolooware Bay, Australia (Evans et al., 2017; Lionel et al., 2013; Martinez-Garcia et al., 2020).

Herpesvirus latent infection can be reactivated when the hosts experience stresses, such as high temperature, immunosuppression, and other infections. Experimentally, herpesvirus reactivation from latency can be induced by immune suppression (Alfaro et al., 2023; Kampouri et al., 2023) and histone chromatin modification (Danaher et al., 2005; Hopcraft et al., 2018). Sodium butyrate (NaB), a histone deacetylase inhibitor, can lead to histone hyperacetylation and turn dormant heterochromatin to euchromatin, and can induce HSV-1 reactivation from latency in mouse models (Neumann et al., 2007). TPCA-1 is an NF-kB innate immune pathway inhibitor and can block inflammatory responses (Larner-Svensson et al., 2010). In the presence of NaB and TCPA-1, the injected oysters started to shed virus again into the seawater within 2 days post-stress (Fig. 1), and viral DNA could be detected in tissues from 2 out 6 stressed oysters (Table 2). Similarly, NaB or TPCA-1 alone could also induce OsHV-1 reactivation from latency at room temperature in naturally infected oysters that survived a mortality event. The OsHV-1 genome, absent in the unstressed oysters, was detected in the gills of 2 out 3 oysters treated with NaB or in the hemocytes of 1 out 3 oysters treated with TPCA-1 within 24 h post-treatment (Fig. 8). Our study suggests OsHV-1, like many other herpesviruses, can become latent and be reactivated under stress conditions. Interestingly, OsHV-1 reactivation only occurs in 30–60 % of those stressed oysters. OsHV-1 latent infection could explain the recurrent infection of OsHV-1 reported by other researchers. For example, an OsHV-1 mortality event was reported in Pacific oysters in seawater that had been UV-treated for up to 64 days at low temperatures after they were briefly exposed to a higher temperature (Pernet and Petton, 2015). In another example reported by Lionel et al. (2013), Pacific oysters that survived an OsHV-1 field mortality event experienced another OsHV-1 mortality event approximately two months later after the temperature was increased. In this second case, it is interesting to note that OsHV-1 was not reactivated when the temperature was changed from approximately 5 °C to approximately 20 °C; rather, OsHV-1 was only reactivated approximately two months later after incoming seawater temperatures reached 16 °C (Lionel et al., 2013). This suggests that elevated temperatures do not always lead to reactivation of OsHV-1; other factors may be involved in OsHV-1 reactivation from latency.

No detectable shedding of OsHV-1 was observed upon experimental injection of two-year-old Pacific oysters with OsHV-1, compared to one-year-old oysters, which might have been due to the increased tolerance of older oysters to OsHV-1 (Hick et al., 2018). Nevertheless, OsHV-1 was detected in tested tissues and hemocytes on 1, 2, and 3 dpi, with higher levels of OsHV-1 in the hemocytes (Fig. 2). During lytic infection in Pacific oysters, OsHV-1 predominately infects hemocytes, which undergo degeneration and death (Bueno et al., 2016; de Lorgeril et al., 2018; Delisle et al., 2022; Friedman et al., 2005). OsHV-1 has also been detected in epithelial cells of Pacific oysters in some studies (Arzul et al., 2002; Martenot et al., 2016; Prado-Alvarez et al., 2016) but not in others (Bueno et al., 2016; Corbeil et al., 2015; Lipart and Renault, 2002), suggesting that epithelial cells are not the primary sites of replication during lytic infection by OsHV-1. It is unusual for hemocytes to be the primary sites of replication during lytic infection by herpesviruses, since epithelial cells are primary sites of replication during lytic infection for most vertebrate herpesviruses and most fish alloherpesviruses (Aurelian, 1990; Cohen, 2020; Hanson et al., 2011). The tropism of OsHV-1 to hemocytes suggests their use in OsHV-1 detection assays could provide increased sensitivity.

In this study, viral DNA was isolated by treating the hemocytes first with hypotonic buffer followed by centrifugation to reduce contamination of viral DNA with host chromosomal DNA (Jin et al., 2004). Viral DNA is much smaller than chromosomal DNA and can leak out of cells in a hypotonic solution. Viral DNA in the supernatant of the post-hypotonic treatment can be separated by centrifugation from the chromosomal DNA trapped inside the cellular nuclei. This preparation likely significantly decreased the amount of host DNA, which has been shown to act as a PCR inhibitor when present in high concentrations (Fu et al., 2009). The DNA isolated from the hypotonic buffer was mostly viral DNA and fragmented cellular DNA; therefore, its concentration was relatively low, often less than 10 ng/µl from 1 to 2 ml of undiluted hemocyte sample. However, 5 µl to 10 µl of the DNA extract was enough to detect OsHV-1 by nested PCR (Figs. 2B, 3, and 5). OsHV-l DNA was also detectable in the total DNA isolated from the cell pellet of the post-hypotonic treatment; however, the detection rate was lower than in supernatant-derived DNA. This is likely due to interference of the PCR with chromosomal DNA. Hemocytes have been previously found to contain more OsHV-1 copies than mantle tissue during experimental infections (Picot et al., 2022; Schikorski et al., 2011), although conversely, Evans et al. (Evans et al., 2017), using commercial kits to co-extract host and viral DNA, did not find that total DNA extracted from hemolymph was more sensitive for OsHV-1 detection than total DNA extracted from gill tissue.

OsHV-1 DNA could be detected in hemocytes at 21 dpi using only nested PCR and not with the qPCR of the OIE protocol, which suggests that only a very small number of hemocytes contained OsHV-1 DNA (Figs. 2 and 3). This finding is similar to those for other herpesviruses that become latent in immune cells; for example, koi herpesvirus (KHV) is latently present in 0.6 % of koi peripheral white blood cells (Reed et al., 2015), and cytomegalovirus (CMV) is latently present in 0.004–0.01 % of human mononuclear cells (Slobedman and Mocarski, 1999). On the other hand, hemocytes may not be the only place that OsHV-1 becomes latent. Other researchers have found that OsHV-1 DNA and proteins were not restricted to a particular tissue or cell type in asymptomatic juvenile and adult oysters (Arzul et al., 2002; Martenot et al., 2016), which suggests that there might be more cell types capable of being latently infected with OsHV-1. There is precedence for a herpesvirus to infect different cell types latently; for example, varicella–zoster virus is latently present in 2–5 % of human neurons as well as <0.1 % of nonneuronal cells (Kennedy et al., 1998).

The DNA polymerase gene (ORF100), considered an early gene, and transmembrane genes (e.g., ORF88), usually expressed later, are typically expressed during lytic infection. However, the mRNAs of ORF100 and ORF88 were also detectable in hemocytes at 21 dpi and oysters sampled 9 months after surviving an OsHV-1 mortality event in Tomales Bay (Figs.4 and 6). The detection of mRNA from genes expressed during lytic infection at 21 dpi suggests that OsHV-1 latency might be similar to CMV latency, where genes expressed during latency are similar to those expressed late in its lytic state (Shnayder et al., 2018). Other herpesviruses, such as HSV-1, have usually been thought to transcribe only one or a few genes during latency, but a growing body of literature suggests that lytic gene transcription is not uncommon in latent cells (Singh and Tscharke, 2020). Depending on how many transcripts OsHV-1 produces for a particular gene per latently infected cell, RT-PCR might be a more sensitive method for detecting latent infections than PCR. In addition, latency-associated transcripts have been identified in many herpesviruses studied, such as LAT of HSV, CLTs of CMV, LMPs of EBV, and ORF6 of KHV (Jenkins et al., 2004; Perng et al., 1994; Qu and Rowe, 1992; Renzaho et al., 2019; Sample and Kieff, 1990; Zwaagstra et al., 1991). Latency-associated transcripts are generally made from unique long inverted repeats. The latency-associated transcript of OsHV-1 is likely encoded in the unique long inverted repeat that may have a similar function as LAT of HSV-1 or ORF6 of KHV. OsHV-1 LAT may be a better strategy for screening oysters with OsHV-1 latent infections since LAT is abundantly expressed during latency.

## 5 Conclusions

In summary, OsHV-1 latent infections were demonstrated in experimentally infected oysters using an OsHV-1 isolate from Tomales Bay, California. Like other herpesviruses, OsHV-1 can also be reactivated and shed into the seawater from surviving oysters. OsHV-1 latency and reactivation from latency were also shown in Tomales Bay oysters sampled 9 and 12 months, respectively, after surviving a mortality event. OsHV-1 latent infections were not detectable in tissues with the qPCR assay recommended by OIE; however, OsHV-1 DNA was detected using a nested PCR. The latency of OsHV-1 was detected with greater sensitivity when DNA samples were isolated from hemocytes treated with a hypotonic buffer. This more sensitive OsHV-1 detection method could be used to screen for latent OsHV-1 in Pacific oysters when viral tissue concentrations are low.

## CRediT authorship contribution statement

**Konstantin Divilov:** Conceptualization, Methodology, Investigation, Data curation, Writing – original draft, Writing – review & editing, Funding acquisition. **Xisheng Wang:** Methodology, Validation, Investigation. **Alexandra E. Swisher:** Investigation. **Peyton C Yeoman:** Investigation. **Maxwell Rintoul:** Investigation, Resources. **Gary B. Fleener:** Methodology, Investigation, Resources. **Blaine Schoolfield:** Methodology, Investigation, Writing – review & editing. **Chris Langdon:** Resources, Writing – review & editing, Funding acquisition. **Ling Jin:** Conceptualization, Methodology, Software, Formal analysis, Resources, Data curation, Writing – original draft, Writing – review & editing, Visualization, Supervision, Project administration, Funding acquisition.

## Declaration of Competing Interest

The authors declare no conflict of interest

## Data Availability

Data will be made available on request.

## Appendix Supplementary materials

Image, application 1
