# Supplementary figures and images for: Ostreid herpesvirus 1 latent infection and reactivation in adult Pacific oysters, Crassostrea gigas
Source: Virus Res. 2023 Oct 25;339:199245. doi: 10.1016/j.virusres.2023.199245 (PMC10613911; doi:10.1016/j.virusres.2023.199245)

## Slide 1
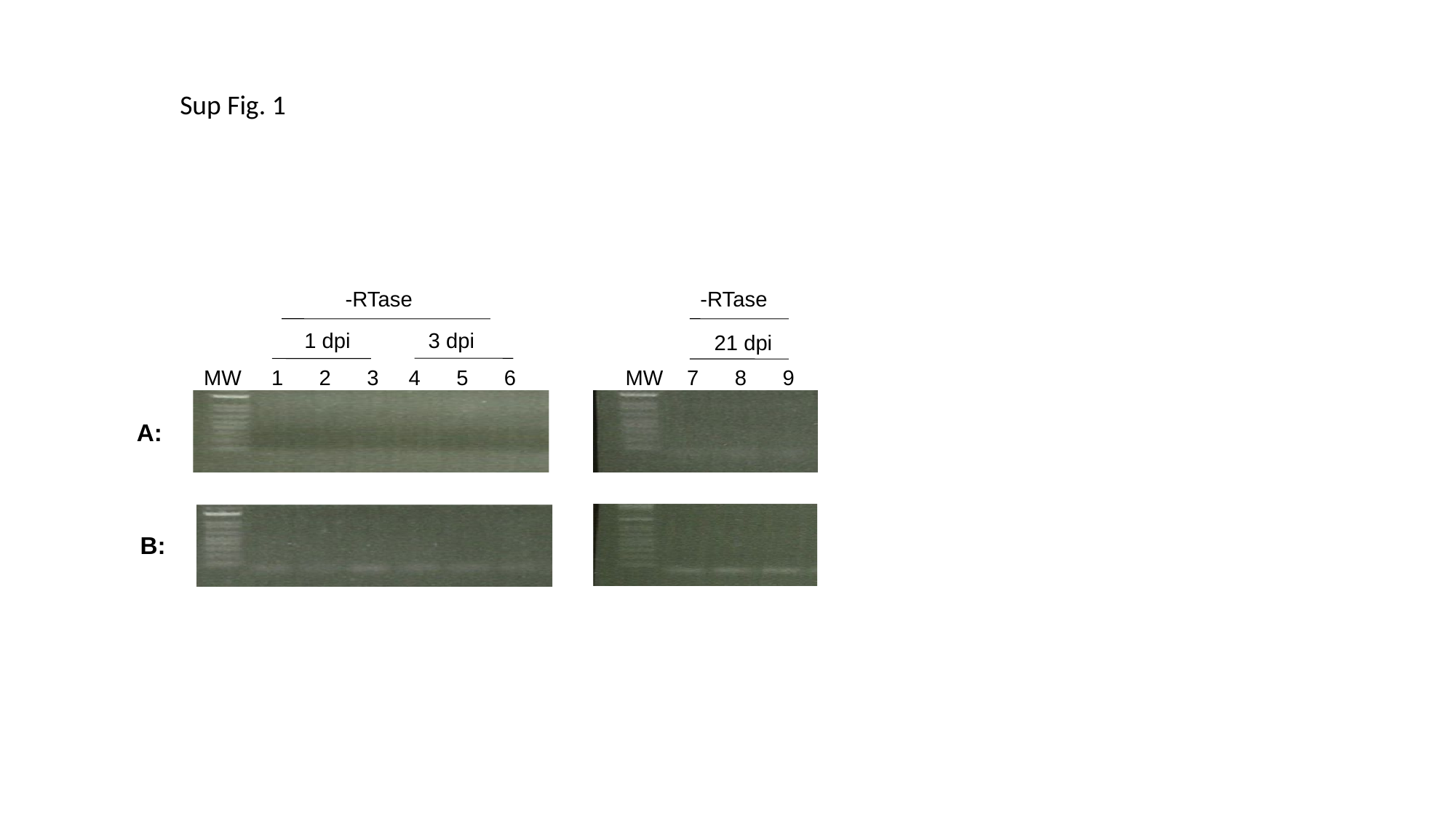

Sup Fig. 1
-RTase
-RTase
1 dpi 3 dpi
21 dpi
MW 1 2 3 4 5 6
MW 7 8 9
A:
B:

Supplement: Supplementary file 1 [file mmc1.ppt]
